# A twenty-first century perspective on concepts of modern epidemiology in Ignaz Philipp Semmelweis’ work on puerperal sepsis

**DOI:** 10.1007/s10654-022-00871-8

**Published:** 2022-04-29

**Authors:** Andreas Stang, Fabian Standl, Charles Poole

**Affiliations:** 1 Institut für Medizinische Informatik, Biometrie und Epidemiologie, Universitätsklinikum Essen, Essen, Germany; 2 School of Public Health, Department of Epidemiology, Boston University, Boston, USA; 3 Department of Epidemiology, Gillings School of Global Public Health, University of North Carolina, Chapel Hill, USA

**Keywords:** Cross infection/history, Cross infection/prevention & control, Cross infection/transmission, Female, Hand disinfection, History, Nineteenth century, Humans, Obstetrics/history*, Pregnancy, Public health/history, Puerperal infection/history*, Puerperal infection/prevention & control

## Abstract

We aimed to review Semmelweis’s complete work on puerperal sepsis mortality in maternity wards in relation to exposure to cadavers and chlorine handwashing and other factors from the perspective of modern epidemiological methods. We reviewed Semmelweis' complete work and data as published by von Györy 1905 according to current standards. We paid particular attention to Semmelweis's definition of mortality in and of itself, to concepts of modern epidemiology that were already recognizable in Semmelweis's work, and to bias sources. We did several quantitative bias analyses to address selection bias and information bias from outcome measurement error. Semmelweis addressed biases that have become known to modern epidemiology, such as confounding, selection bias and bias from outcome misclassification. Our bias analysis shows that differential loss to follow-up is an unlikely explanation for his results. Bias due to outcome misclassification would only be relevant if misclassification differed between time periods. Confounding by health status was likely but could not be quantitatively addressed. Semmelweis was aware that cause-specific mortality is a function of incidence and prognosis. He reasoned in potential outcome terms to estimate the reduced number of deaths from an intervention. He advanced a hypothesis of clinic overcrowding as a risk factor for puerperal sepsis mortality that turns out to be wrong. Semmelweis’ data provide a great pool for illustrating the logic of scientific discovery by use of the numerical method. The explanatory power of his work was strong and Semmelweis was able to refute several previous causal explanations.

**Supplementary Information:**

The online version contains supplementary material available at 10.1007/s10654-022-00871-8.

## Introduction

Sepsis is defined as the presence of infection along with features of systemic inflammation characterized by criteria that define the systemic inflammatory response syndrome. Sepsis that follows childbirth is called puerperal sepsis (PS), puerperal fever or childbed fever. Alexander Gordon in 1795 (Aberdeen) [1] and Oliver Wendell Holmes in 1843 (Boston) [2] suspected that PS is transmitted by the birth attendant, whether midwife or obstetrician. In a series of studies in the General Hospital of Vienna in the mid-1800s, Ignaz Philipp Semmelweis showed that women attended by clinicians who had conducted autopsies were at elevated risk of PS mortality and that chlorine washing of the hands markedly reduced this increased risk. He attributed these results to “cadaverous particles” [3] [pg. 64] about 30 years before Louis Pasteur cultured Streptococcus pyogenes from the blood of a woman who suffered from PS [4]. Semmelweis’ epidemiologic approach was not well received at the General Hospital of Vienna, where the medical school was leading a revolution in anatomical pathology [5].

The controversies spawned by Semmelweis’ theory, before the publication of his famous work in 1861 [6], and his role in the history of medicine have been well documented [7–10]. Statistical aspects of his research have also been discussed [11, 12]. Our aims are quite specific: to provide an accounting of his data that is as complete and accurate as possible and to compare his epidemiologic concepts and methods with those of today.

## Methods

We extracted annual and monthly mortality data from Semmelweis’ 1861 publication [13] that is reprinted in [3]; reviewed the German monograph, published in 1905 by von Györy, reprinted in [3], and translated into English by Murphy in [14]. The monograph includes notifications by Hebra, Routh, Haller and Skoda in 1848 of results from Semmelweis’ studies. We identified numerical discrepancies within and between these sources, which we attempted to resolve and correct in a comprehensive data set in the online Supplement, which also provides specific references to page numbers and table numbers for all extracted data and discrepancy resolutions.

The epidemiologic concepts and methods we applied to Semmelweis’ data are described in the Results section. Today’s names for the concepts are given in italics. References to page numbers in the 2007 reprint of von Györy’s 1905 monograph [3] are given in brackets.

## Results

### Source population, numerator and denominator of Semmelweis’ mortality data

The first maternity clinic at the General Hospital of Vienna opened in 1784. In 1833, a second maternity clinic opened. Beginning in 1840, medical students and junior physicians were educated in Clinic 1, midwives in Clinic 2. Semmelweis studied women admitted to these clinics from 1784 to 1858. Equating the number of hospitalized women with the number of births occurring in the clinics, we estimate that the two clinics accounted for 34% of all births in Vienna in 1840–1850 [15] (Supplementary Table 1).

No woman had PS on admission. Many were admitted while pregnant and either gave birth to live infants, experienced in-hospital abortions or stillbirths, or died in-hospital while still pregnant. Other women were admitted after giving birth to live infants or after experiencing abortions or stillbirths outside of the hospital. The numbers in these categories were not recorded. All we know is how many women were admitted in a given time period and how many died of PS in that period. Semmelweis most likely assigned the month or year of death to the month or year of admission respectively (Supplementary Text 1). Thus, the PS percentages Semmelweis calculated were not risks, but akin to today’s *pregnancy-related maternal mortality ratio (PRMR)*, for which the numerator is the number of pregnancy-related maternal deaths in a geographic areas in a given time period and the denominator is the number of live births in that area in the same time period [16]. Hence, it would be more accurate to call the frequency measure Semmelweis calculated a puerperal sepsis mortality ratio (PSMR), rather than a risk. Semmelweis’ PSMRs are also similar to the *infant mortality “rates”* that are calculated today as the number of infant deaths divided by the number of live births in jurisdictions in which birth and death files have not yet been linked [17]. Healthy inpatient women were discharged no later than day 9 [pg. 411] and the mean length of hospital stay was not reported by Semmelweis so that the mortality risk period is not well-defined.

### Development of PSMR from 1784 through 1858

Semmelweis was aware that *mortality is a function of incidence and prognosis*. He stated that the higher mortality at Clinic 1 was due to a higher incidence of PS and claimed that the probability of recovery and death were the same in the two clinics [pg. 64, 119]. He clarified that the *relative mortality* (i.e. *risk*) and not the *absolute mortality* (i.e. *absolute number of deaths*) needs to be studied as the absolute mortality in a small clinic as in Würzburg (Bavaria) cannot become as large as in a large clinic (as Vienna) [pg. 262, 330, 367].

Our re-analysis of the annual mortality data reveals that, in the first 39 years of the maternity clinic (1784–1822), the PSMR was low (median PSMR 0.8%). Only two out of 39 years had PSMRs above 3.0% (1814: 3.2%, 1819: 5.0%). With the introduction of pathological anatomy in 1823, the PSMR (for Clinic 1 and Clinic 2 together) increased up to 12.1% (1842) and thereafter decreased. The lowest annual PSMR after 1842 in both clinics combined was in 1848 (1.3%) when chlorine washing of the hands was taking place in Clinic 1 (Supplementary Fig. 1).

**Fig. 1:**
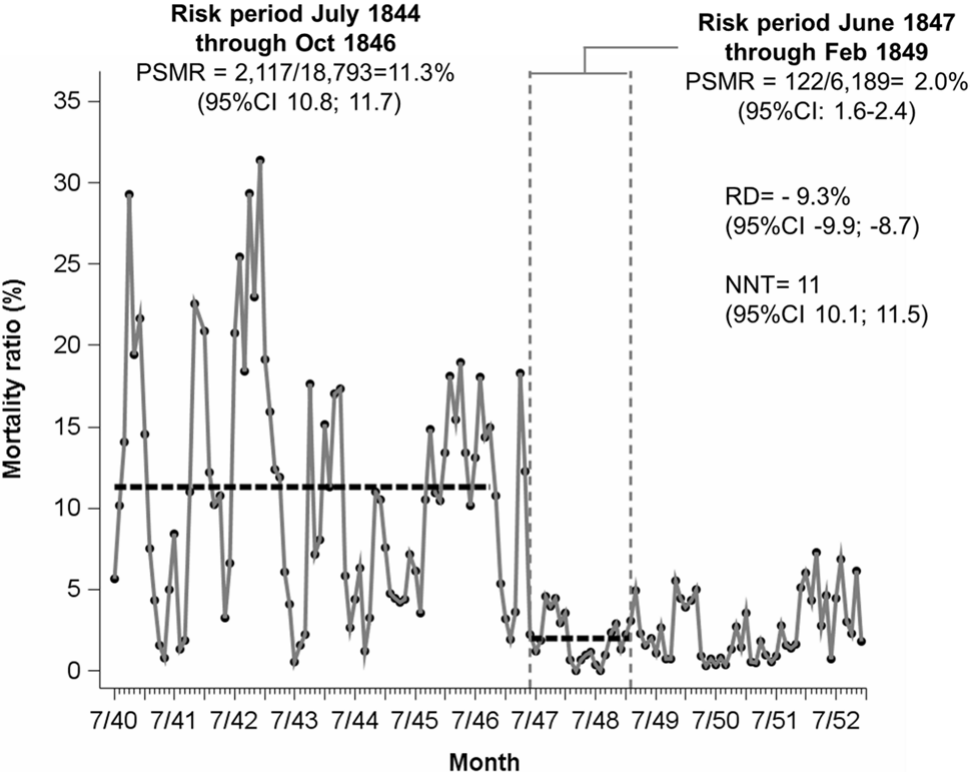
Recalculated monthly mortality ratios in Clinic 1, General Hospital Vienna (July 1840 through January 1853). Legend: first vertical reference line (June 1847): the first full month of chlorine washing of the hands; second vertical reference line (Feb 1849): the last full month of chlorine washing before Semmelweis leaves the Clinic; RD: difference of mortality ratios, 95%CI: 95% confidence interval; NNT: number needed to treat for the comparison of the later period with the earlier period; the average number of puerperae per months was 265

Our re-analysis of monthly mortality data shows that in Clinic 1, before the chlorine handwashing, the PSMR was 11.3% (2117/18,793) in July 1840 through October 1846. In November 1846 through March 1847, before handwashing was introduced, Semmelweis noticed that the PSMR in Clinic 1 declined to 4.9% (75/1,523). This pre-intervention decline presented a challenge to Semmelweis hypothesis. He explained it by a change of the first assistant physician. Semmelweis who regularly performed autopsies of deceased women was assistant physician at Clinic 1 from July 1st, 1846 through October 20th, 1846. Thereafter, assistant physician Breit became first assistant physician. During Breit’s presence at Clinic 1, he and his medical students were rarely undertaking or joining autopsies [pg. 40; pg. 141]. On March 20th, 1847, Semmelweis became again first assistant physician at Clinic 1. Beginning in April 1847, the first full month when Semmelweis became the first assistance through May 1847, the PSMR was elevated once again to 18.3% (57/312) in that month. In the next month during which Semmelweis recalled beginning with handwashing the PSMR was 12.2% (36/294). These changes of the first assistant physicians (and therefore the change of the autopsy-related potential to transmit PS from deceased women to puerperae) resemble an early *de-challenge re-challenge* phenomenon [pg. 140]. After introduction of chlorine washing (mid May 1847), the PSMR during the period June 1847 through February 1849 in Clinic 1 was 2.0% (122/6,189) (Fig. 1).

**Fig. 2:**
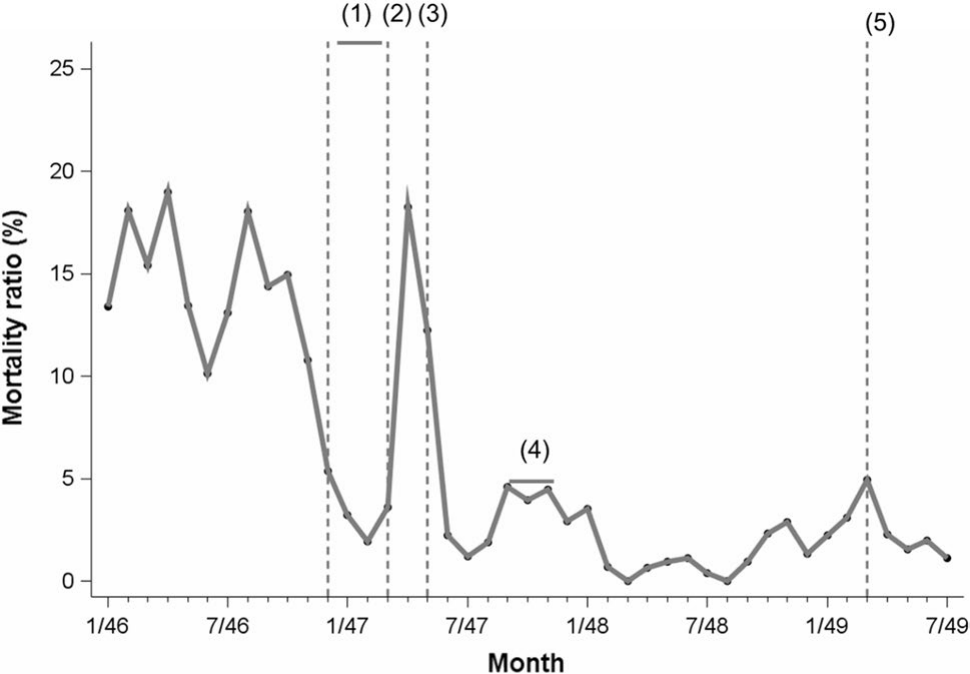
Recalculated monthly mortality ratios (%) at Clinic 1, General Hospital, Vienna, January 1846 through July 1849. Legend: (1) December 1846 to March 1847: first assistant Breit and his students rarely undertake or join autopsies; (2) Semmelweis becomes first assistant again (March 20, 1847); (3) Mid of May 1847: start of chlorine washing of the hands; (4) September 1847: lack of compliance of chlorine hand washing by medical students and junior physicians, October 1847 treatment of a puerpera with a foully discharging medullary carcinoma of the uterus, November 1847 treatment of a puerpera with discharging carious left knee; (5) Semmelweis leaves Clinic 1 on March 20, 1849

The PSMR rapidly went down to 3% in July 1847. The lack of careful hand washing of medical students and junior physicians (September 1847) [pg. 267], the omission of hand washing after the examination of a women with a foully discharging medullary carcinoma of the uterus (October 1847) [pg. 165], and the examination of a puerperae with a discharging “carious knee” [pg. 165] explained the temporarily higher mortality risk (4.0–4.6%) according to Semmelweis (Fig. 2).

### Estimability of various measures of effect

**Fig. 3:**
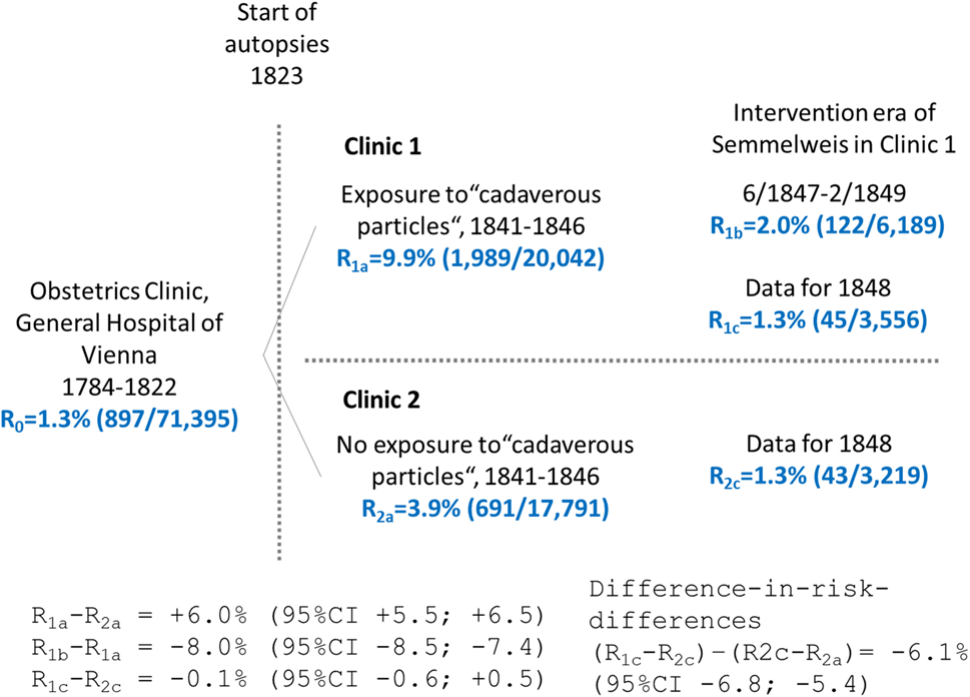
Recalculated mortality ratios of different time periods and exposure potential to “cadaverous particles”. Legend R: indicates mortality ratio; numbers in parenthesis indicate deaths and inpatient women of the corresponding time periods respectively

Under ideal circumstances, similarly as in a *cohort study* (and not case–control study as previously claimed about Semmelweis’s data [10, 18], several risk difference measures (RD) with 95% confidence intervals (95%CI) based on PSMRs can be estimated:

- R_1a_‒R_2a_ compares Clinic 1 in 1841–1846, before the introduction of handwashing, with Clinic 2, also with no handwashing, in the same period;
- R_1b_‒R_1a_ compares Clinic 1 in the handwashing period (June 1847 through February 1849) with the same clinic before the introduction of handwashing (1841–1846);
- R_1c_‒R_2c_ compares Clinic 1 with Clinic 2 in the only complete calendar year of handwashing in Clinic 1 (1848); and
- (R_1c_‒R_1a_) ‒ (R_2c_‒R_2a_) estimates the difference-in-risk-difference (Fig. 3).

### Seasonality of PSMR in Clinic 1

At Semmelweis’ time, the Galenic theory (epidemic constitution and its atmospheric-cosmic-telluric conditions) was a popular etiologic theory to explain the seasonal occurrence (preponderance in the winter) of PS. However, Semmelweis explained this seasonality by varying numbers of medical students at Clinic 1 who had to be educated: During summer vacation, often a third of the students as compared to the winter period were only present [pg. 55–56; pg. 170]. Even more important, in the winter, surgical exercises with cadavers were performed before the 4:00 p.m. ward round. In the summer, these exercises were performed in the evening after the 4:00 p.m. ward round due to the heat [pg. 55–56; pg. 170]. That is, Semmelweis had an alternative explanation why the PSMR followed a seasonal pattern.

**Fig. 4:**
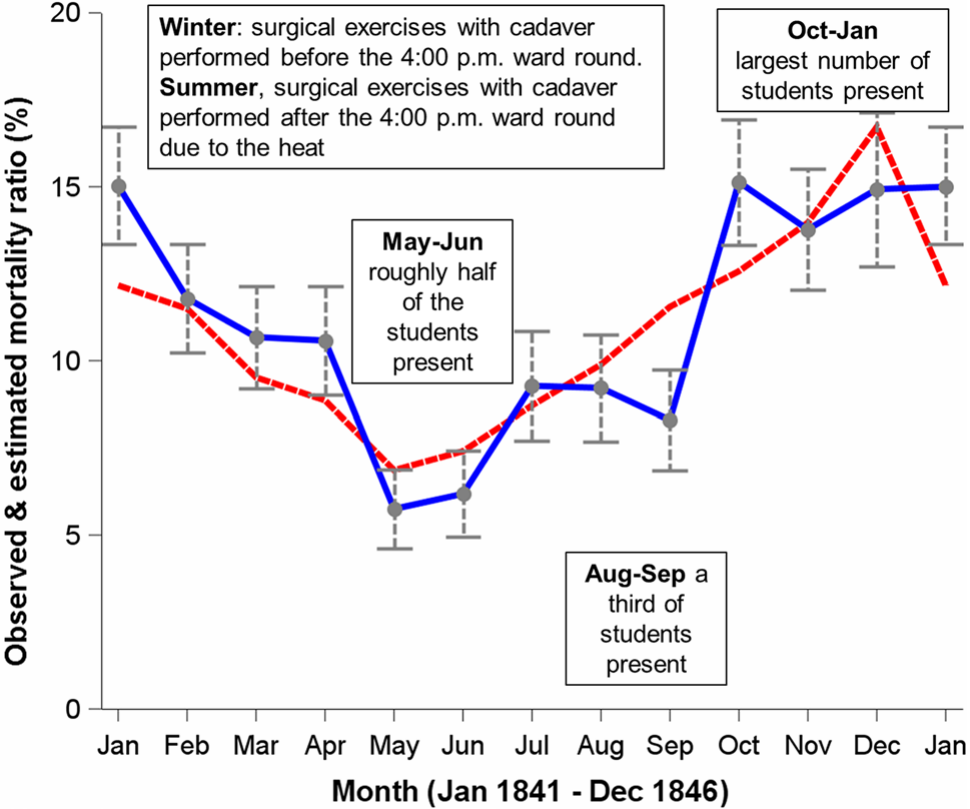
Seasonality and mortality ratio at Clinic 1, General Hospital Vienna, July 1840 through November 1946. Legend: vertical whiskers indicate 95% confidence intervals; the blue line indicates the monthly observed mortality ratio and the red dotted line indicates the monthly estimated mortality ratios; estimated seasonal relative risk 2.0 (95%CI: 1.8–2.3) with an estimated time of peak on December 14 (344°)

To estimate the intensity of seasonal occurrence of deaths in Clinic 1, we collapsed PSMRs of the same months for the period January 1841 to December 1846 and used an estimator of the intensity of seasonal occurrence. This estimator is based on the assumption of a single cyclical effect (harmonic) that can be well approximated by a sine curve [19]. Our estimated peak/low ratio also called the seasonal relative risk was 2.0 (95%CI: 1.8–2.3). The estimated time of peak was December 14th (344°) (Fig. 4).

### Puerperal sepsis of the newborn

Semmelweis noted that also newborns at Clinic 1 had a higher sepsis mortality. He found that newborns of women with PS did not acquire sepsis at the same time as their mothers [pg. 157] and therefore, the infection of newborns occurred postpartal and not in utero. He concluded that the infection of newborns is due to placental or postpartal transmission of infectious particles from the mother to the child. Newborns who died of sepsis had very similar pathological findings as women who died of PS. He concluded that chlorine washing of the hands had the same effect on PS risk among newborns as among puerperae. The medical report about Semmelweis’ discovery by Haller of 1848, published in Semmelweis collected works, includes annual death statistics of the newborns. Our re-analysis of the newborn mortality ratio in Clinic 1 and 2 revealed that for the period 1841–1846, before the introduction of handwashing in Clinic 1, the mortality ratio among newborns was 7.6% in Clinic 1 and 3.8% in Clinic 2 (RD = 3.9%, 95%CI 3.4; 4.3). In 1848, the only full year when the medical staff in Clinic 1 did chlorine hand washing, the mortality ratio among newborns was 4.2% in Clinic 1 and 3.2% in Clinic 2 (RD = 1.0%, 95%CI 0.1; 2.0). The difference-in-risk-difference was −2.8% (95%CI −3.9; −1.9) (Supplementary Fig. 2).

### Bias from differential loss to follow-up

Haller [pg. 35] and Skoda [pg. 36] explained that a substantial number of ill women from Clinic 1 and Clinic 2 were transferred to other wards of the General Hospital for humanitarian and sanitarian reasons when the number of PS cases became excessive. Semmelweis stated that deaths of women who finally died on these wards could not be recorded by him. The transfer policy to other wards differed by Clinic. Semmelweis stated that during times of excessive PS mortality, “all ill women with” PS were transferred en masse from Clinic 1 to other wards of the General Hospital and transfers of this extent were never made from Clinic 2. Only sporadic women were transferred away from Clinic 2 [pg. 100–101]. Semmelweis concluded that the difference of the PS mortality between Clinic 1 and Clinic 2 of the period 1841–1846 was actually considerably larger than reported (*biased effect estimate*) [pg. 100–101]:

“*The difference in mortality between the two Clinics, as great as this table shows it to be, was in truth much greater, because at times due to the increasing mortality … all ill puerperae were transferred from Clinic 1 to the General Hospital, died there, and then were entered in the returns of the General Hospital as deaths, but not in those of the Lying-in Hospital*” [p 100].

In May 1841, 255 women were admitted to Clinic 1 and Semmelweis estimated that 60–80 women (24–31%) were transferred to other wards than maternity wards in the General Hospital [pg. 369]. Importantly, Semmelweis stated that since June 1847, that is the month after he started with chlorine washing, ill women were not transferred to other wards of the General hospital anymore (with the exception of women with syphilis, cow pox etc.) [pg. 52].

Our bias analysis revealed that even if this transfer of ill women from Clinic 1 to other wards had continued to some extent during the intervention period, the number of women who would have to be transferred to artificially produce the observed mortality decline after the introduction of chlorine washing in 1847 had to be substantial. In 1848, 45 from 3556 women died at Clinic 1 (1.3%) and 43 from 3219 women died at Clinic 2 (1.3%). To produce a mortality ratio at Clinic 1 in 1848 as in the year 1846 (11.4%) would require that additional 360 women from Clinic 1 who eventually died (that is the eightfold number of the actually reported 45 deaths in Clinic 1 in 1848) were transferred to other wards than maternity wards in the General Hospital. We consider such a magnitude unrealistic.

### Outcome misclassification bias

Semmelweis noted that not every death of a woman was due to PS as caused by autopsy-related cadaverous particles [pg. 164]. However, he used the overall mortality ratio as a *proxy measure* of the PS-specific mortality ratio. He once reported figures that allow the calculation of PS deaths as a proportion of all deaths for the period September 1840 to September 1842 (92%, [pg. 366]) (for details see Supplementary Text 1). Thus, from the point of view of possible misclassification, the sensitivity of outcome classification was 100%, while the specificity was less than 100% because some proportion of deaths were not attributable to PS. We ran a deterministic sensitivity analysis to study outcome misclassification scenarios. The observed PSMR difference of − 8.0 percentage points is used as the starting value (PSMR difference R_1b_-R_1a_, see Fig. 3) [20]. We varied the specificity of outcome classification (70–100%) in both periods. Only misclassification scenarios in which specificity ranged between 91 and 100% resulted in the prediction of positive numbers of deaths and thus, meaningfully, corrected RD estimates. The corrected RD estimates only differed markedly if misclassification was differential by period and the range of corrected RD estimates was −8.9 to + 1.0 percentage points (Supplementary Table 2). Semmelweis did not provide any evidence that outcome misclassification would be time-dependent. However, such a change would be necessary to justify the differential misclassification scenario shown above (specificity in the early period markedly lower than in the late period) that could explain a RD bias of the magnitude of about 8 percentage points, if the true RD would be zero. There are numerous scenarios that may explain the time dependency of misclassification, e.g. change in staff, qualification and experience, however, we have not found any evidence that can be used to identify a specific scenario.

### Potential confounding by health status

The Vienna maternity clinic was a “free maternity ward”, which meant that women were treated for free [pg. 99]. However, women who were treated in Clinic 1 of the Vienna hospital had to agree on participating in obstetrical exercises for junior doctors and for the training of midwives.

It appears that women had no choice and were distributed to the clinics according to the fixed algorithm as described by Semmelweis. In Clinic 1, women were not be admitted from Tuesday 4:00 p.m. to Wednesday 4:00 p.m., from Thursday 4:00 p.m. to Friday, 4:00 p.m. and from Sunday, 4:00 p.m. to Monday 4:00 p.m. During these periods, women were admitted only to Clinic 2 [pg. 61–62; pg. 99–100]. Semmelweis reported that Clinic 1 had developed a bad reputation among the women. Viennese women were afraid to be admitted to Clinic 1 [pg. 118] and they tried not to report for admission during the periods when they knew they would be admitted to Clinic 1 [pg. 62, 332]. Consequently, Semmelweis noted that the health status of the women in Clinic 1 was generally much less favorable [pg. 332]. The information Semmelweis gave about the poor reputation of Clinic 1 enables us to conjecture that women aware of that reputation and in good health were more able to ensure that they would go to Clinic 2 and avoid Clinic 1. This systematic allocation of women to the two Clinics (and the circumvention of this allocation by healthier women) clearly created the *bias* that he documented that nowadays we would call *confounding*. To control this confounding would require measures of health status at admission which was not recorded by Semmelweis.

### Semmelweis’ reporting of a key moment on the way to the "cadaverous particle" hypothesis

We have compiled a list of historical data as reported by Semmelweis that led him to his discovery as presented in Semmelweis's collected works (Supplementary Table 3). Semmelweis stated that he had no clue regarding the high mortality in Clinic 1 during his first appointment as first assistant (July 1846 to October 1846) and the list of alternative explanations as presented in his collected works was long (Supplementary Table 4).

Semmelweis changed the birth position in Clinic 1, which had been horizontal, to lateral, as in Clinic 2, because that initially appeared to him be the only material difference between the two clinics [pg. 129]. Later, Semmelweis clarified a crucial difference between the Clinics: midwife pupils in Clinic 2 were not allowed to join autopsies and did not get in contact with ill puerperae as they were not allowed to join the ward round [pg. 373–374]. Within a few months only, Semmelweis came up with the hypothesis that “cadaverous particles” from autopsies at the hands of obstetricians were the transmitters of PS in Clinic 1 and introduced chlorine washing of the hands in the mid of May 1847. Semmelweis states that this idea was triggered by the death of Prof. Kolleschtka on March 13, 1847 who died from septicemia (very similar pathological findings as among PS deaths) due to a laceration from a scalpel wielded by a medical student during an autopsy [pg. 129–130].

### Semmelweis’ animal experiments [pg. 143–146] and early causality aspects

Semmelweis cited Josef Hamernik who long before Robert Koch described *causal conditions* that have to be fulfilled for establishing a causal relation between an exposure and an outcome: (1) the exposure must always have the same consequence, (2) the outcome can always be experimentally produced by the exposure, and (3) if the outcome cannot be experimentally produced by the exposure, the reason for each failure has to be found out [pg. 350]. Semmelweis argued that cadaverous particles are a *necessary* but not *sufficient cause* for PS; not every rabbit that Semmelweis experimented with died of pyemia. In relation to condition 2 and 3, Semmelweis only stated that he had been able to produce the disease among rabbits but was unable to explain why some rabbits did not get pyemia; however, he added a fourth causal condition, that is, the experimental removal of the risk factor, i.e., cadaveric particles by chlorine washing of the hands should reduce the mortality risk.

### Exposure dose and PSMR

Semmelweis observed that nearly all women who suffered from a lengthening of the first stage of birth (shortening and dilatation of the uterine cervix) in Clinic 1 subsequently developed PS. The same lengthening of the first stage among women in Clinic 2 was “harmless” [pg. 121–122]. Semmelweis estimated that women whose first stage of birth lasted at least three days in Clinic 1 underwent at least 30 vaginal examinations (10 per day) [pg. 304] and therefore these women had a substantial *exposure potential* for cadaveric particles. He considered the first stage of birth as the most vulnerable period for the acquisition of PS [pg. 125]. He also concluded that the second stage (descent of the child) is not a vulnerable period for transmission of PS anymore, as contact with vulnerable surfaces of the uterus during this stage is not possible [pg. 122].

Furthermore, some thousand women [pg. 67] who were not able to reach the General Hospital in time self-delivered in the streets of Vienna annually (called street births). Many of these women nevertheless went to the General Hospital as their children would be admitted to a foundling home for free. These women had to agree that they participated in the education of medical students and junior physicians in Clinic 1. However, according to Semmelweis, these women were not of interest for teaching purposes because the placenta of these women was usually expulsed when they arrived in Clinic 1 [pg. 139] (*lack of exposure potential*). Unfortunately, Semmelweis did not stratify his mortality data by place of birth (streets or in-hospital) as he did not consider this stratification as important [pg. 125]. Finally, women with premature birth had a lower PSMR as they had a lower probability of being examined for teaching purposes [pg. 129].

### Association between overcrowding of the maternity clinics and PSMR

According to Semmeweis, it was hypothesized by Scanzoni [pg. 334], Martin [pg. 386], and Arneth [pg. 522] among others that overcrowding in the clinic was responsible for PS. To illustrate that overcrowding of Clinic 1 was not associated with PSMR, Semmelweis tabulated his monthly mortality figures of Clinic 1 sorted by season and the number of women admitted to Clinic 1 [pg. 108–109] and found that a decreasing amount of overcrowding was not accompanied with a decreasing PSMR.

To study the association between crowding and PSMR, we used three time periods and both Clinics: (1) monthly PSMRs during the period July 1840 to April 1847 of Clinic 1 were potentially biased because of en masse transfer of ill women to other wards of the General Hospital and therefore underascertainment of deaths (see above); (2) monthly PSMRs during the period June 1847 to December 1852 of Clinic 1 when chlorine handwashing took place in Clinic 1 and during which the en masse transfer of ill women did not take place anymore so that underascertainment of death did not play a relevant role anymore; (3) the period January 1849 to December 1852 of Clinic 2, where en masse transfer never took place.

**Fig. 5:**
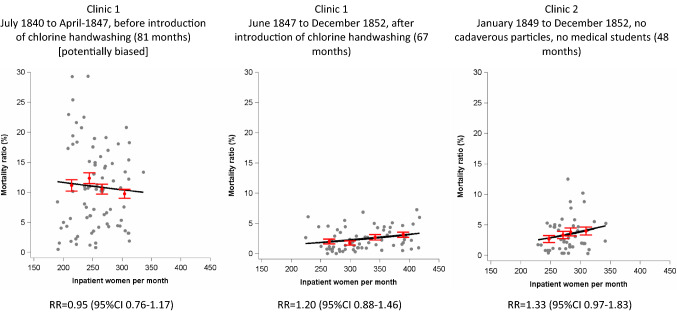
Association between monthly number of women in Clinic 1 and 2 and puerperal sepsis mortality ratio (%). Legend : Relative risk of puerperal sepsis death per increment of 50 inpatient women per month; Red points and whiskers indicate point estimates with 95% confidence intervals per quartile of inpatient women per month; monthly mortality ratios from July 1840 to April 1847 in Clinic 1 were potentially biased because of en masse transfer of ill women to other wards of the General Hospital; trend lines from log-binomial models with empirical sandwich errors

Figure 5 displays scatterplots of the monthly number of women and monthly PSMRs for the three time periods. To address overdispersion, we ran log-binomial models with empirical sandwich errors to estimate relative risks per increment of 50 women for the association between monthly number of inpatients and monthly PSMRs. During the first period in Clinic 1 (biased data), we found a negative association with an estimated RR of 0.95 (95%CI 0.76–1.17). During the second period of Clinic 1 (unbiased) and in Clinic 2, we found a positive association (Fig. 5). In this case, Semmelweis seems to have been mistaken.

### Epistemological aspects

Semmelweis made frequent use of the *modus tollens* or the valid argument in propositional logic of denying the consequent (premise 1: if A then B, premise 2: not-B, conclusion: not-A) to refute several alternative causal explanations of PS at his time. For example, if atmospheric or telluric influences (A) cause PS (B), and the mortality risk in Clinic 2 is considerably lower than in Clinic 1 (quasi not-B), atmospheric or telluric influences (A) cannot be an explanation, given everything else the same (*ceteris paribus*).

Semmelweis used *counterfactual reasoning* to estimate the number of puerperal deaths that would not (sic) have been prevented if chlorine washing of the hands had not been introduced. This *estimated caseload reduction* was 837 over a period of 6 years in Clinic 1 [pg. 391–392]. He believed in the ubiquitous validity of laws of nature and therefore in the applicability of his theory also to Würzburg, where one of his major opponents (Prof. Scanzoni) worked (*generalizability, external validity, transportability*) [pg. 263, 360, 414].

Our detailed comparison of presented statistical data in Semmelweis [13], von Györy [3], and Murphy [14] showed that Murphy’s English translation of Semmelweis’s publications contains too many errors and should not be used for statistical re-analyses of Semmelweis’ data. Examples of errors are provided in the Supplementary Text 2.

## Discussion

Semmelweis’ mortality studies provide a great pool for illustrating the logic of scientific discovery by use of the “medical arithmetic tradition” in eighteenth century Britain [21, 22] and the “numerical method” in nineteenth century France [23]. However, Semmelweis did not quote Louis’ work although Louis’ work was already described in German text books on the history of medicine in 1843 [24]. Semmelweis’ studies that included mortality data of 261,725 women and 10,294 deaths from 1784 to 1858 is one of the first large scale numeric studies in clinical medicine.

Many concepts of modern epidemiology are already recognizable in Semmelweis’ work including mortality as a function of incidence and prognosis, the preference of relative mortality (PSMR) over absolute numbers of deaths for comparison purposes, the concept of de-challenge and re-challenge, biases due to selection, outcome misclassification, and confounding, potential outcome reasoning, dose–response concepts, and generalizability. Our deterministic bias analyses show that Semmelweis’ results are relatively robust with respect to selection bias and information bias. Unfortunately, Semmelweis did not characterize the difference in health status between women in Clinic 1 and 2, so a bias analysis with respect to confounding was not possible.

Five key features enabled Semmelweis to come up with the correct explanation of the biomedical cause of PS: (1) the introduction of pathological anatomy and therefore autopsy of patients—a major source of exposure to cadaveric particles—at the General Hospital of Vienna, one of the largest maternity clinics of the world in 1823 [25], (2) the foundation of two separate maternity clinics in the General Hospital in 1833, one with this source of exposure, the other without it, (3) the similarity of the pathological findings after Prof. Kolletschka has been autopsied, (4) the systematic tabulation of the number of hospital admission of women and the number of in-hospital deaths among these women from 1784 through 1858, and finally (5) the decline in PSMR after removal of the exposure to cadaverous particles by chlorine hand washing.

The failure to convince opinion-leading obstetricians and academic physicians at Semmelweis’ time may have had several reasons: (1) he could only conclude that contaminations (cadaveric particles) of the hand may be the cause of PS. The germ theory of PS had been proposed some 30 years later. Acceptance of Semmelweis’ discovery therefore meant that the two prevailing theories of the etiology of PS, miasmatic theory (“a bad component in the air”) and atmospheric-cosmic-telluric conditions (Galenic hypothesis), had to be denied without the introduction of a new pathology-based theory; (2) the numerical method in medicine as introduced by Louis was still in its infancy [26]; (3) Semmelweis delayed the publication of his work until 1861 (13 years delay); (4) Semmelweis’ style of scientific writing was wordy and repetitious [7, 27, 28], and (5) the style of communication that Semmelweis used to defend his discovery of PS transmission was harsh and personally offensive [10, 29].

In one of his open letters, Semmelweis summarized his view of the cause of PS: every case of PS (*necessary cause*) had contact to a deleterious (or decaying) agent that was resorbed; this agent is transmitted either by the finger of the examiner, the hand of the surgeon, instruments, bed linen, atmospheric air, sponges, or by the hands of midwives that got in contact with the agent from severly ill puerperae or other severly ill patients [pg. 453]. The explanatory power of his work was strong and Semmelweis was able to refute several previous causal explanations as nobody before him was able to do. Given the scientific *Zeitgeist* and statistical methods available, the authors of this article cannot help but join historians Carter [9] and Newsom [10] in seeing Semmelweis as a pioneer of epidemiology.

## Supplementary Information

Below is the link to the electronic supplementary material.

Supplementary file1 (XLSX 28 kb)

Supplementary file2 (DOCX 412 kb)
