# Supplementary material for: A twenty-first century perspective on concepts of modern epidemiology in Ignaz Philipp Semmelweis’ work on puerperal sepsis
Source: Eur J Epidemiol. 2022 Apr 29;37(5):437–45. doi: 10.1007/s10654-022-00871-8 (PMC9209376; doi:10.1007/s10654-022-00871-8)
Supplement: Supplementary file 2 — Supplementary file2 (DOCX 412 kb) [file 10654_2022_871_MOESM2_ESM.docx]

**Supplementary material**

**A 21st century perspective on concepts of modern epidemiology in Ignaz Philipp Semmelweis’ work on puerperal sepsis**

Table of Contents

[Suppl. Table 1: Hospitalization of puerperae in Vienna 2](#_Toc98081484)

[Suppl. Table 2: Sensitivity analysis of outcome misclassification 4](#_Toc98081485)

[Suppl Table 3: Selected historical data of puerperal sepsis 5](#_Toc98081486)

[Suppl. Table 4: Alternative hypotheses about the etiology of puerperal sepsis 7](#_Toc98081487)

[Suppl. Table 5: Statistical figures of 1841 by Herzig 10](#_Toc98081488)

[Suppl. Table 6: Bibliographic details: von Györy T, 1905 12](#_Toc98081489)

[Suppl. Figure 1: Annual mortality ratios of the maternity clinic, 1784-1858 14](#_Toc98081490)

[Suppl. Figure 2: Newborn mortality ratios in Clinic 1 and Clinic 2 15](#_Toc98081491)

[Supp. Text 1: Details about Semmelweis’ mortality data 16](#_Toc98081492)

[Suppl. Text 2: Data errors 18](#_Toc98081493)

[Suppl. Text 3: Miscallenous 20](#_Toc98081494)

[References (Supplement) 21](#_Toc98081495)

# Suppl. Table 1: Hospitalization of puerperae in Vienna

**Fraction of puerperae in Vienna that was hospitalized in Clinic 1 and 2**

*Period 1801-1825 (before Semmelweis practiced in the hospital)*

Schmidt reported that from 1801 through 1825 (25 year period) the average number of births per year was 12,055 (1). During the period 1801 through 1825, Semmelweis was not working at the hospital. However, Semmelweis reported for the same period a total of 53,716 puerperae in Clinic 1 and 2. We equaled the number of pueperae hospitalized with the number of births. That means, on average, the number of puerperae per year in the Clinics was 2,149. Therefore, the proportion of puerperae seen in the Vienna Clinics (1801-1825) was 2,149/12,055 = 18%.

*Period 1840-1850 (period during which Semmelweis temporarily practiced in the hospital)*

Weigl reported the annual number of births in Wien of the years 1840 through 1850 (2). We used Semmelweis annual numbers of women who were admitted to Clinic 1 or Clinic 2 during the same period. Again, we equaled the number of hospitalized women with the number of births. By dividing the number of births in the hospital through the number of births in Vienna, we estimated the percentage of births in the Vienna hospital. Overall, the estimated percentage of births was 34% (**Supplementary Table 1**).

**Supplementary Table 1**

**Women admitted in Clinic 1 and 2 and births in the Vienna hospital and in Vienna overall**

| **Year** | **Admitted women, Clinic 1 & 2 (Semmelweis)** | **Births in Vienna (Weigl)** | **Percentage** |
| --- | --- | --- | --- |
| 1840 | 5,166 | 18,277 | 28 |
| 1841 | 5,454 | 16,571 | 33 |
| 1842 | 6,024 | 18,047 | 33 |
| 1843 | 5,914 | 17,984 | 33 |
| 1844 | 6,244 | 18,524 | 34 |
| 1845 | 6,756 | 19,206 | 35 |
| 1846 | 7,027 | 19,811 | 35 |
| 1847 | 7,039 | 19,019 | 37 |
| 1848 | 7,095 | 18,915 | 38 |
| 1849 | 7,229 | 19,516 | 37 |
| 1850 | 7,006 | 20,121 | 35 |
|  |  |  |  |
| 1840-1850 | 70,954 | 205,991 | 34 |

# Suppl. Table 2: Sensitivity analysis of outcome misclassification

**Sensitivity analysis of bias from imperfect specificity of puerperal sepsis mortality ascertainment before and during the handwashing period in Clinic 1**

|  | **False positivity (%) during the handwashing period (%)** | |
| --- | --- | --- |
| **False positivity (%) before the handwashing period** | 0 | 1 |
| 0 | -8.0 | -8.9 |
| 1 | -7.0 | -8.0 |
| 2 | -6.1 | -7.1 |
| 3 | -5.2 | -6.2 |
| 4 | -4.2 | -5.2 |
| 5 | -3.2 | -4.2 |
| 6 | -2.2 | -3.2 |
| 7 | -1.2 | -2.2 |
| 8 | -0.1 | -1.1 |
| 9 | +1.0 | 0.0 |

The sensitivity is assumed to be 100%. The observed data were 1,989 PS deaths among 20,042 women before handwashing was introduced (1841-1846) and 122 PS deaths among 6,689 women with clinician handwashing (June 1847 to February 1849) (see R1_b_-R1_a_, Figure 3).

# Suppl Table 3: Selected historical data of puerperal sepsis

**Selected historical data of puerperal sepsis at the maternity wards, General Hospital of Vienna, 1784-1857, as reported by Semmelweis**

| **Date** | **Historical date** |
| --- | --- |
| August 16th, 1784 | Opening of the maternity clinic at the General Hospital (Allgemeines Krankenhaus) of Vienna, Austria; puerperae do not pay fee for service. |
| Since 1823 | Start of autopsies of the deceased; pathological anatomy introduced into the teaching curriculum for medical students in Vienna [pg. 70, 203] |
| 1833 | Foundation of a second maternity clinic at the General Hospital of Vienna with education of medical students, junior physicians and midwives at both clinics [pg. 99, 182] |
| October 27th, 1840 | Governmental act, Z. 61015: [pg. 99, 134-135], that separates the education of male medical students and junior physicians from female pupil midwives by teaching the former group in Clinic 1 only and the latter group in Clinic 2 only [pg. 182] |
| July 1st, 1846 to October 20th, 1846 | First appointment of Semmelweis as first assistant (lecturer) of Clinic 1 [pg. 97]; Semmelweis eagerly undertakes autopsies |
| October 20th, 1846 to March 20th, 1847 | Appointment of Dr. Breit as first assistant (lecturer) of Clinic 1; from December 1846 through March 1847, he and his students rarely joined or undertook autopsies [pg. 40, 141] |
| Since March 20th, 1847 | Second appointment of Semmelweis as first assistant (lecturer) of Clinic 1 [p. 97]; Semmelweis eagerly undertakes autopsies [pg. 40] |
| March 13th, 1847 | Professor Kolletschka, professor of forensic medicine, died from septicemia after laceration from a scalpel wielded by a medical student during an autopsy; Semmelweis read the report of Kolleschka’s autopsy and was struck by the similarity of the pathological findings to puerperae who had died of PS [pg. 129-130] |
| Mid of May 1847 | Semmelweis introduces chlorine washing of the hands [pg. 65] |
| September 1847 | Lack of compliance of chlorine hand washing by medical students & junior physicians [pg. 267] |
| October 1847 | A puerpera was examined who had a „foully discharging medullary carcinoma“ of the uterus without hand disinfection by chlorinated lime; as a consequence, 11 of the 12 puerperae delivered along with her died [pg. 165] |
| November 1847 | A puerpera with a discharging carious left knee was examined and not isolated [pg. 165] |
| March 20th, 1849 - Sommer 1853 | Carl Braun, opponent of Semmelweis‘ theory, becomes first assistant after Semmelweis‘ departure (March 20, 1849- Summer 1853) [pg. 183] |
| April 1853 – December 1857 | Gustav Braun (April 1853 – December 1857): opponent of Semmelweis‘ theory, becomes first assistant after Carl Braun [pg. 183] |

# Suppl. Table 4: Alternative hypotheses about the etiology of puerperal sepsis

**Alternative hypotheses about the etiology of puerperal sepsis described and discussed by Semmelweis**

| **Competing etiologic hypothesis** | **Semmelweis’ reaction & counterarguments** |
| --- | --- |
| ***Internal factor theories*** |  |
| Hyperinose (excessive fibrin in the blood], hydremie [excessive water in the blood], plethora [excessive quantity of blood], disturbances caused by the pregnant uterus, stagnation of the circulation, inopexia [spontaneous coagulation of blood], delivery itself, decreased pressure, protracted labor, wounding of the inner surface of the uterus, imperfect contractions, faulty involution of the uterus after birth, scanty and discontinued secretion and excretion of lochia, suppression of milk secretion, death of the fetus, individuality of the patient [pg. 64, 119, 121] | these conditions must be equally harmful or harmless in both clinics;  these conditions should occur in both clinics with the same prevalence [quasi-randomized hospital admission];  it does not explain, why newborns of PS puerperae at Clinic 1 died more often due to sepsis  it does not explain why chlorine washing of the hands reduced the mortality risk of PS |
| Anxiety of being admitted to Clinic 1 [pg. 118] | it does not explain why chlorine washing of the hands reduced the mortality risk of PS |
| Sperma virile and metamorphoses [pg. 121] | all women became pregnant by male sperms; does not explain, why newborns puerperae with PS at Clinic 1 died more often due to sepsis; it does not explain why chlorine washing of the hands reduced the mortality risk of PS |
| Hypothermia, dietary causes [pg. 120] | both wards were essentially identical in terms of layout and infrastructure; it does not explain why chlorine washing of the hands reduced the mortality risk of PS |
| Hurt sense of shame [pg. 119] | it does not explain why chlorine washing of the hands reduced the mortality risk of PS; it does not explain, why newborns of PS puerperae at Clinic 1 died more often due to sepsis |
| ***External factor theories*** | **Semmelweis’ reaction & counterarguments** |
| Overcrowding of Clinic 1 [pg. 63, S. 105] | empirically falsified  it does not explain why chlorine washing of the hands reduced the mortality risk of PS |
| Poor air ventilation of Clinic 1 [pg. 120] | Both wards were essentially identical in terms of layout and infrastructure  it does not explain why chlorine washing of the hands reduced the mortality risk of PS |
| Contaminated walls, furnitures, beds, chairs and linen [pg. 63, S. 120] | it does not explain why chlorine washing of the hands reduced the mortality risk of PS |
| Miasmic theory (noxious vapors and smells as causes) | did not explain why the much more crowded Clinic 2 had lower mortality risks; does not explain, why newborns of PS puerperae at Clinic 1 died more often due to sepsis |
| Galenic theory (epidemic constitution and its atmospheric-cosmic-telluric conditions) | Implausible, as both clinics had an identical atmosphere; does not explain, why newborns of PS puerperae at Clinic 1 died more often due to sepsis |
| Liebigian theory (non-living organic substances conveying disease) | Semmelweis’ favorite hypothesis |
| Crude manual obstetric examinations by medical students in Clinic 1 [pg. 63, 119] | it does not explain why chlorine washing of the hands reduced the mortality risk of PS |
| Transfer from labor room to childbed through a cold hallway [pg. 120] | it does not explain why chlorine washing of the hands reduced the mortality risk of PS |
| Different treatments at Clinic 1 and 2 [pg. 119] | the necessity to apply obstetric surgery or maneuvers was essentially the same in Clinic 1 and 2 [quasi-randomized hospital admission] and was done by the same physicians |
| Foreign students as a cause of PS | after reduction of foreign students, the mortality at Clinic 1 showed barely any change |
| Priest walking around the clinics preceded by a Sacristan ringing a bell to administer the last rites | Semmelweis managed to still the ringing without effect; does not explain, why newborns of PS puerperae at Clinic 1 died more often due to sepsis |

**Legend Supplementary Table 2:**

Compiled alternative etiologic hypothesis as presented in Semmelweis's collected works; we used the terminology “internal factor theories” and “external causation theories” from Daniels, 1998 (3).

# Suppl. Table 5: Statistical figures of 1841 by Herzig

**Statistical figures of 1841 related to Clinic 1 reported by Herzig, as orally delivered to him by Dr. Zipfel and Dr. Lumpe (4)**

| **Characteristic** | **Number** |
| --- | --- |
| Number of pregnant women hospitalized in Clinic 1 | 2,882 |
| Number of living births | 2,749 |
| Number of still births | 128 |
| Early miscarriages month 3 or 4 of pregnancy | 5 |
| Number of women who gave birth and were discharged | 2,588 |
| Number of women who did not give birth and were discharged | 47 |
| Number of children discharged | 2,550 |
| Women who died | 238 |
| Children who died | 179 |

For the same year (1841), Semmelweis reported a total of 3,036 women hospitalized and 237 puerperal deaths. The number of hospitalized women differs by 154 women who were counted by Semmelweis but not by reported by Zipfel and Lumpe. A potential reason for this difference is the statistical treatment of admitted women who were transferred to wards other than maternity wards in the General Hospital of Vienna. For example, in May 1841, 255 puerperae were admitted to Clinic 1 and Semmelweis estimated that 60-80 puerperae (24-31%) were transferred to other wards than maternity wards in the General Hospital [pg. 369].

The number of puerperal deaths in 1841 differs only by one woman (Semmelweis: n=237, statistic: n=238). Semmelweis stated that the Vienna hospital admitted pregnant women before delivery, in labor, and some women after birth [pg. 99]. Semmelweis obviously also treated women at Clinic 1 who did not give birth during the hospital stay (and obviously also not before the hospitalization as street birth). Furthermore, he treated women with an early miscarriage (month 3 or 4 of pregnancy). However, the data which Semmelweis collected were restricted to women who gave live birth, still birth or early abort either just before hospitalization (“street births”) or during the hospital stay [pg. 100]. Semmelweis considered a stratification of the mortality data by place of delivery (street versus Clinic 1) not as important (“fühlte ich das Bedürfnis einer solchen Tabelle nicht”) [pg. 125].

# Suppl. Table 6: Bibliographic details: von Györy T, 1905

**von Györy T (Ed). Semmelweis’ Gesammelte Werke. Budapest 1905. Reprint 2017. VDM Verlag Dr. Müller e.K. und Lizenzgeber. Saarbrücken 2007 (5)**

| **Pages** | **Title of the report*** |
| --- | --- |
| 1-18 | **Semmelweis IP**: Tractatus de vita plantarum (dissertation inauguralis) (orig. Latin) |
| 23-25 | **Hebra F**: Höchst wichtige Erfahrungen über die Aetiologie der in Gebäranstalten epidemischen Puerperalfieber (1847-1848) |
| 25-33 | **Routh CHF**. Ueber die Ursachen des endemischen Puerperalfiebers in Wien (1848) (orig. English) |
| 34-35 | **Haller C**. Aerztlicher Bericht über das k. k. allgemeine Krankenhaus in Wien und die damit vereinigten Anstalten: die k. k. Gebär-, Irren- und Findel-Anstalt im Solar-Jahre 1848 |
| 36-45 | **Skoda J**. Ueber die von Dr. Semmelweis entdeckte wahre Ursache in der Wiener Gebäranstalt ungewöhnlich häufig vorkommenden Erkrankungen der Wöchnerinnen und des Mittels zur Verminderung dieser Erkrankungen bis auf die gewöhnliche Zahl (1848) |
| 49-51 | Aus dem Protokoll der allgemeinen Versammlung der k. k. Gesellschaft der Aerzte zu Wien vom 15. Mai 1850 |
| 51-53 | Aus dem Protokoll der allgemeinen Versammlung der k. k. Gesellschaft der Aerzte zu Wien vom 18. Juni 1850 |
| 54-58 | Aus dem Protokoll der allgemeinen Versammlung der k. k. Gesellschaft der Aerzte zu Wien vom 15. Juli 1850 |
| 61-83 | **Semmelweis IP**. Die Aetiologie des Kindbettfiebers (1858) |
| 83-94 | **Semmelweis IP**. Der Meinungsunterschied zwischen mir und den englischen Aerzten über das Kindbettfieber (1860) (orig. Hungarian) |
| 95-426 | **Semmelweis IP**. Die Aetiologie, der Begriff und die Prophylaxe des Kindbettfiebers (1861) |
| 427-440 | **Semmelweis IP**. Zwei offene Briefe an Dr. J. Spaeth und an Hofrath Dr F. W. Scanzoni |
| 441-462 | **Semmelweis IP**. Zwei offene Briefe an Hofrath Dr. E. C. J. v. Siebold und an Hofrath Dr. F. W. Scanzoni |
| 463-511 | **Semmelweis IP**. Offener Brief an sämmtliche Professoren der Geburtshilfe |
| 512-537 | Der Verein St. Petersburger Aerzte über die Aetiologie und die prophylactische Behandlung des Kindbettfiebers (1863) (orig. Hungarian) |
| 540-597 | **Semmelweis**‘ gynaecologische Aufsätze (orig. some of them in Hungarian) |

Legend: *If the language of the original publication was other than German, the original language is indicated; Quotations or page numbers from Semmelweis' writings always refer to this German language work and page numbers are indicated in parenthesis [pg. xx] in this document.

# Suppl. Figure 1: Annual mortality ratios of the maternity clinic, 1784-1858

**
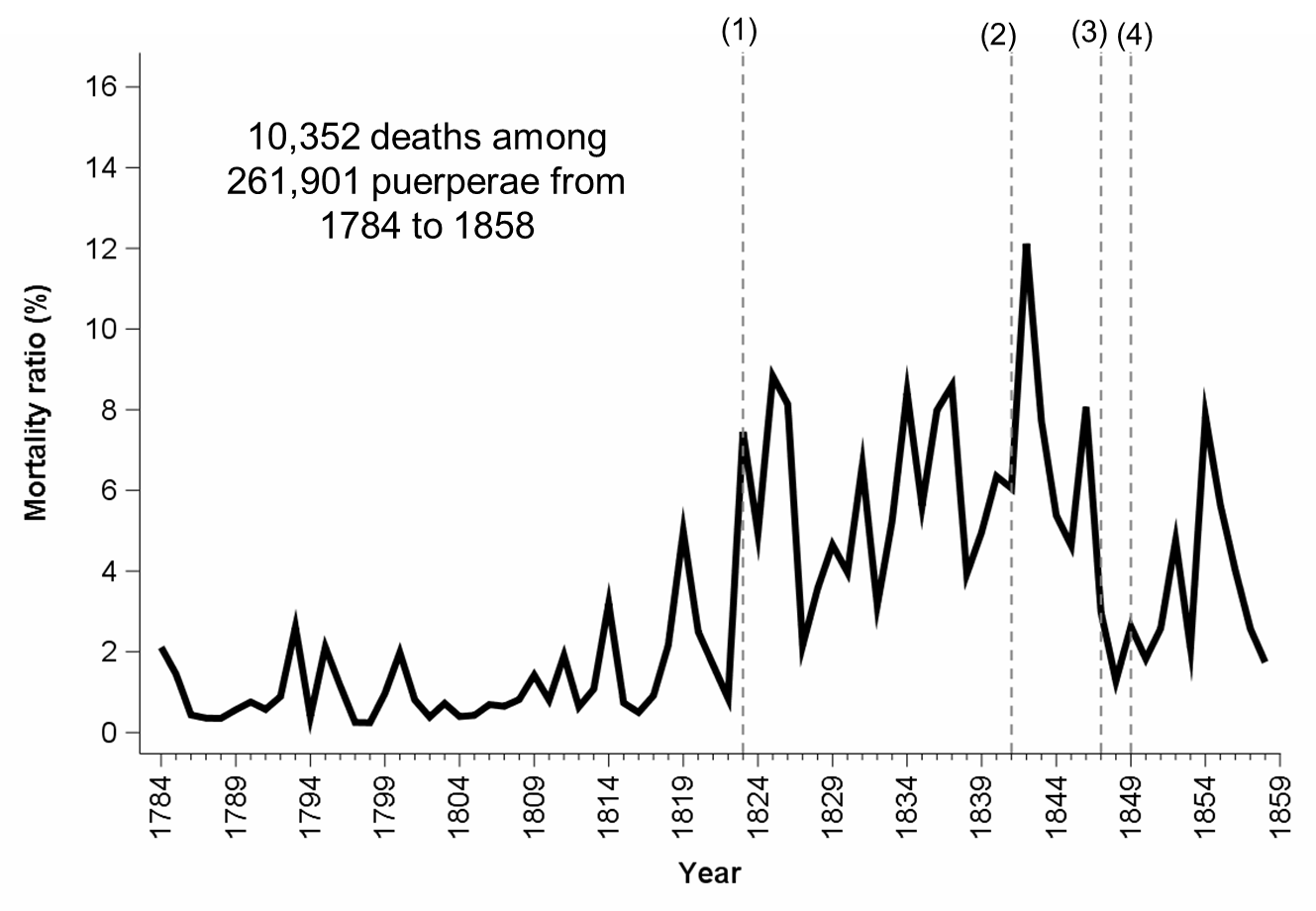
**

**Legend:**

(1) 1823: start of pathological anatomy; (2) 1841: medical students and physicians are only taught at Clinic 1, pupil midwives are only taught at Clinic 2; (3) 1847: introduction of chlorine washing of the hands by Semmelweis; (4) Semmelweis leaves Clinic 1 on March 20, 1849; Carl and thereafter Gustav Braun became first assistant at Clinic 1 and were opponents of Semmelweis’ theory; re-analysis and visualization by the authors.

# Suppl. Figure 2: Newborn mortality ratios in Clinic 1 and Clinic 2

**
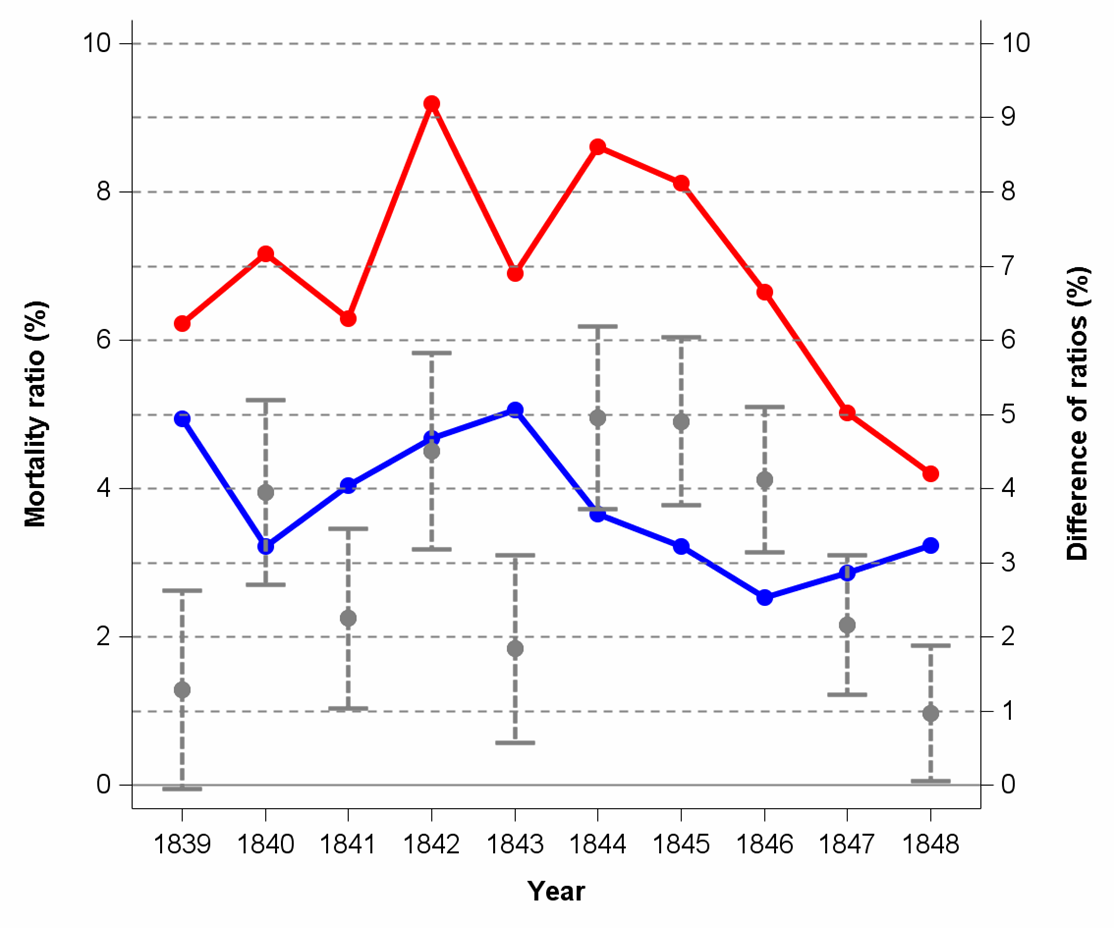
**

Legend: percentage of newborn deaths in Clinic 1 (red) and Clinic 2 (blue); in 1848 (more exactly May 1847 through March 1849), chlorine washing of the hands was applied in Clinic 1; difference of mortality ratios (Clinic 1 minus Clinic 2) and 95% confidence intervals: data from Haller’s report about Semmelweis’s discoveries; re-analysis and visualization by the authors. The differences of mortality ratios for the period 1841-1846 was 3.9% (95%CI 3.4-4.3) and for the year 1848 was 1.0% (95%CI 0.1-0.2);

# Supp. Text 1: Details about Semmelweis’ mortality data

*Semmelweis’ reporting period*

Semmelweis reported annual mortality statistics (PSMRs) from 1784 to 1858 of the maternity clinics of the General Hospital of Vienna. In addition, he reported annual mortality statistics for Clinic 1 and Clinic 2 separately for the years 1833 through 1858. Furthermore, he reported monthly mortality statistics for the period July 1840 through December 1852 for Clinic 1 (with the exception of December 1841 because Semmelweis lost these data [pg. 103]) and for a narrower period of April 1849 through December 1852 for Clinic 2. Semmelweis did not exactly define the mortality risk in terms of counting which deaths, assignment of deaths to calendar dates, population at risk and risk period.

*Assignment of calendar date for date of death*

From his writings one can indirectly conclude that he assigned the calendar date of hospital admission to deaths occurring thereafter: “November 1841 starben 53 Wöchnerinnen von 253 Wöcherinnen, also 22,55%” (English translation: “In November 1841, 53 pueperae from 253 puerperae died, that is 22.55%”) [pg 103]. Semmelweis furthermore spoke of the “number of deceased in relation to the number of women taken care of” [pg. 164, Table XVIII].

*Population at risk*

Semmelweis mentioned the hospitalization of pregnant women before delivery, in labor, and some women after birth (“Schwangere, Kreissende, Wöchnerinnen”) [pg 99]. Herzig (1848, (4) ([pg. 79-80] reported statistical figures of Clinic 1 of the year 1841. With a footnote, he explains that the figures were orally reported by two physicians working in Clinic 1, Dr. Zipfel and Dr. Lumpe (**Supplementary Table 5**).

*Denominator of the mortality statistics*

Semmelweis used several wordings in the many statistical tables throughout his writings and obviously always meant women who just gave birth: “Wöchnerin” [latin: puerpera, woman who just gave birth], “Geburten” [engl. births], “Individuen” [engl. individuals], “Gebärende” [latin: puerpera], “Entbundene” [engl. women who gave birth]; all these terms indicate that the denominator is the number of hospitalized women who gave birth, that is, aborts (miscarriage), stillbirths or births alive (either within the hospital or just before, so-called street births); it is not the number of babies born obviously.

Semmelweis also registered women who gave birth in the streets of Vienna just before arrival in the hospital. At page 67, Semmelweis stated that “annually several thousand street births” (“Gassengeburten”) took place in Vienna. “The number of hospitalized women who gave street birth exceeded monthly at both clinics often 100” [pg. 125].

*Mortality risk period*

Semmelweis did not report an average length of hospital stay after delivery. He only stated that “healthy women in childbed had to be discharged on day 9.” [pg. 411]. However, Herzig (4) [pg. 79] reported a usual stay of 9-10 days after delivery in the early 1840ies. Semmelweis suspected that the mortality risk of PS depended on the length of hospital stay by explaining that another maternity ward (“Zahlgebärhaus”), where pregnant women had to pay for treatment, underestimated the mortality risk of PS, as inpatient discharge often occurred a few hours or days after delivery [pg. 173].

*Timing of arrival of women*

Semmelweis did not explicitly state whether women with early abortions were counted in his statistics. According to his use of medical terminology (“Wöchnerinnen”) he most likely only counted women who gave birth (aborts, stillbirths, births alive). He apparently included women who gave birth just before arrival in the hospital (street births). According to Herzig, quoting Zipfel and Lumpe who both were working as physicians in Clinic 1, treatment of pregnant women in Clinic 1 also included early abortions (month 3 and 4) (4).

*Overall mortality as a surrogate for the puerperal sepsis mortality*

It appears that Semmelweis counted any death in his mortality statistics and attributed the deaths to PS. As the number of causes of death during the puerperium is quite small (sepsis, severe bleeding, thrombembolic events), the overall mortality appears to be a good proxy measure of the PS-specific mortality. Semmelweis himself was aware of potential misclassification of cause of death when he talked about the mortality statistics before 1823, the period before introduction of autopsies and therefore low mortality: “this low mortality is maybe not the smallest possible, because singular (sic) puerperae may have died due to other diseases [than PS]” [pg. 164]. Semmelweis noted that not all deceased puerperae died of PS [pg. 73-74]. He considered the proportion of women who died due to other causes than PS as very small. “Some (original: “einzelne”) of the maternity wives who died did not die of childbed fever, but of other diseases.” [pg. 164]. This means that he considered the total mortality as a reasonable surrogate of the PS-specific mortality. In a letter, Semmelweis told Dr. Levy that women who gave birth die only exceptionally from causes other than PS [pg. 275-276] so that total mortality is a good proxy of the PS-specific mortality. Interestingly, when Semmelweis worked at St. Rochus Hospital in Pest in 1855 and had introduced chlorine washing of the hands, he distinguished between deaths due to PS and death by other causes [pg. 148, pg. 154].

# Suppl. Text 2: Data errors

*Data errors in Semmelweis’ tables (1861) (6)*

In Table VI, Semmelweis (page 15) reports a mortality percentage of 30.61 in January 1842 for 64 deaths among 307 puerperae which actually is 20.8%. In Table XVII (page 62), Semmelweis reports 1,574 admitted puerperae in 1792. However, in Table XXXI (page 171) he reports 1,579 puerperae for the same year. In Table XXIV, page 142, Semmelweis incorrectly reports a mortality risk of 3.38% for Clinic 2 before introduction of chlorine washing of the hands. The correct percentage is 3.9% (691/17,791). For the total period 1784 through 1858, Semmelweis (1861) reports in Table XXIV (page 142) 262,523 puerperae and 10,282 deaths. However, when adding up the reported annual figures the total number of puerperae is 261,901 and deaths is 10,352. In Table XXXI (page 172) last row, Semmelweis summarizes the overall number of puerperae and deaths over a period 1784 through 1849 (66 years). He reports 153,841 puerperae and 6,224 deaths. He did not explain that for the years 1833 through 1849, he did not include pueperae and deaths of Clinic 2. The correct total number of admitted puerperae and deaths from 1784 through 1849 is 193,996 puerperae and 7,878 deaths. Semmelweis listed the annual number of women and the number of deaths in Clinic 2 for the years 1841 through 1846 (Table LXV, page 304). He added up these numbers for the total period 1841-1846 and incorrectly reported overall 27,791 puerperae. The correct figure is 17,791.

*Data errors in the German language reprint (von Györy 1905 (5)) and English translation (Murphy 1981 (7))*

Our supplementary data file contains the annual and monthly mortality data that Semmelweis reported. Several of these statistics were also reported on other places than the tables or pages that we cite. We checked von Györy and Murphy’s statistical figures with those of Semmelweis. Interestingly, both the reprint from 1905 (von Györy) and the English translation by Murphy (e.g. page 366) re-printed numerical errors produced by Semmelweis. In addition, they produced additional numerical errors.

*Data errors in the reprint from 1905 (von Györy) (5)*

At page 405, the number of deaths in March 1851 in Clinic 1 is incorrectly reported to be 1 death. However, in the original publication by Semmelweis 1861 (page 510) overall 2 deaths were reported.

*Data errrors in the English translation (Murphy) (7)*

In Table VIII, Murphy (page 370) reported 408 deaths among 209 puerperae in November 1842 (which is statistically impossible). In Semmelweis 1861, this number is 48. In Table XV, Murphy (page 389) reproduced the wrong number of puerperae in February 1847 (n=912) from Semmelweis 1861 who originally produced this typo. The correct number of puerperae should be n=312 and the mortality should be 1.92% as in Semmelweis’ Table III instead of 1.02%. Table XVII, Murphy (page 398) is incorrectly labeled as “Statement of the Imperial and Royal Lying-in Hospital from 16 August, 1784”. However, these data are not from 1784 as indicated by the title of the table but start with the year 1784 as stated by Semmelweis. Furthermore, the first year reported is not 1884 but 1784. In Table XVIII, Murphy (page 436) incorrectly reports 2105 puerperae in 1801. However, the correct number is 2106 according to Semmelweis 1861. In Table XXII, Murphy (page 457) reports the total number of deaths in Clinic 2 for the period 1833-1840 as n=73. However, the correct number is n=731 as reported by Semmelweis 1861. However, Murphy reports the correct percentage of deaths here. In Table XXXI, Murphy (page 486) reports for the year 1829 overall 3,912 puerperae. The correct number according to Semmelweis (1861) is 3012. However, Murphy reports the correct percentage of deaths (4.64%) here. Mortality data for 11/1852, 8/1850, 8/1849, 5/1850, and 6/1849 of Clinic 1 were left out in Murphy 1981, whereas Semmelweis presents these data at page 513. Similarly, mortality data for 11/1852, 8/1850, 8/1849, 5/1850, and 6/1849, Clinic 1 were left out in Murphy 1981, whereas Semmelweis presents these data at page 513.

# Suppl. Text 3: Miscallenous

*Case-fatality*

Semmelweis reported of women who suffered from PS but survived it [pg 74-75]. He noted that the percentage of recovery from PS was identical in Clinic 1 and 2 [pg. 119]: “The cases of recovery among the truly ill women in childbed were not different between the two Clinics” (German: “Die Genesungsfälle unter den wirklich erkrankten Wöchnerinnen waren auf beiden Abtheilungen nicht verschieden”). He concluded that the higher mortality in Clinic 1 is due to the higher risk to acquire PS.

*Acquisition of PS*

Semmelweis believed that PS only occurs in the context of the hospitalization of pregnant women before delivery, in labor, and women after birth [pg. 66]. According to Semmelweis, PS occurred also among pregnant women who were hospitalized before delivery and had to undergo medical teaching rounds in the Clinic [pg. 66, pg. 126]. Nevertheless, Semmelweis considered PS to be a disease that exclusively affects women who have recently given birth [pg. 123], although he also observed PS in pregnant women and spoke of "peculiar blood mixtures" in this context [pg. 129].

*Disinfection methods used by Semmelweis*

Semmelweis first used chlorina liquida. After a while, he changed to the less expensive chlorinated lime which was a good choice from nowadays perspective. A 5 minutes application of this solution reduces the transient bacterial flora of the hands by 5.5 powers of 10, that is down to 1/300,000. This reduction is larger than nowadays hand disinfection with 60% isopropanol for 1 min according to European standards (EN) for products and services 1500 (8).

# References (Supplement)

1. Schmidt A. Wien wie es ist. Die Kaiserstadt und ihre nächste Umgebungen nach authentischen Quellen mit besonderer Berücksichtigung wissenschaftlicher Anstalten und Sammlungen. 2 ed. Wien: Carl Gerold Verlag; 1837.

2. Weigl A. Demographischer Wandel und Modernisierung in Wien. Wien: Pichler Verlag; 2000.

3. Daniels IR. Historical perspectives on health. Semmelweis: a lesson to relearn? J R Soc Promot Health. 1998;118(6):367-70.

4. Herzig W. Das Medicinische Wien. Wegweise für Aerzte und Naturforscher, vorzugsweise für Fremde. 2. edition ed. Wien: Wilhelm Braumüller Verlag; 1848.

5. von Györy T. Semmelweis' gesammelte Werke. Saarbrücken: VDM Verlag; 2007.

6. Semmelweis IP. Die Aetiologie, der Begriff und die Prophylaxis des Kindbettfiebers. Pest: C. A. Hartleben's Verlags-Expedition; 1861.

7. Murphy FP. The etiology, the concept and the prophylaxis of childbed fever together with the "open letter" by Ignac Semmelweis. Birmingham: The Classics of Medicine Library; 1981.

8. Rotter M. Ignaz Philipp Semmelweis. Vater der geburtshilflichen Infektionsprävention. Gynäkologe. 1999;32:496-500.
